# Redistribution of H3K4me2 on neural tissue specific genes during mouse brain development

**DOI:** 10.1186/1471-2164-13-S8-S5

**Published:** 2012-12-17

**Authors:** Jie Zhang, Jeffrey Parvin, Kun Huang

**Affiliations:** 1 The CCC Biomedical Informatics Shared Resource, The Ohio State University, USA; 2 Department of Biomedical Informatics, The Ohio State University, USA

## Abstract

**Background:**

Histone modification plays an important role in cell differentiation and tissue development. A recent study has shown that the dimethylation of lysine 4 residue on histone 3 (H3K4me2) marks the gene body area of tissue specific genes in the human CD4+ T cells and neural cells. However, little is known of the H3k4me2 distribution dynamics through the cell differentiation and tissue development.

**Results:**

We applied several clustering methods including K-means, hierarchical and principle component analysis on H3K4me2 ChIP-seq data from embryonic stem cell, neural progenitor cell and whole brain of mouse, trying to identify genes with the H3K4me2 binding on the gene body region in different cell development stage and study their redistribution in different tissue development stages.

A cluster of 356 genes with heavy H3K4me2 labeling in the gene body region was identified in the mouse whole brain tissue using K-means clustering. They are highly enriched with neural system related functions and pathways, and are involved in several central neural system diseases. The distribution of H3K4me2 on neural function related genes follows three distinctive patterns: a group of genes contain constant heavy H3K4me2 marks in the gene body from embryonic stem cell stage through neural progenitor stage to matured brain tissue stage; another group of gene have little H3K4me2 marks until cells mature into brain cells; the majority of the genes acquired H3K4me2 marks in the neural progenitor cell stage, and gain heavy labeling in the matured brain cell stage. Gene ontology enrichment analysis also revealed corresponding gene ontology terms that fit in the scenario of each cell developmental stages.

**Conclusions:**

We investigated the process of the H3K4me2 mark redistribution during tissue specificity development for mouse brain tissue. Our analysis confirmed the previous report that heavy labeling of H3K4me2 in the downstream of TSS marks tissue specific genes. These genes show remarkable enrichment in central neural system related diseases. Furthermore, we have shown that H3K4me2 labeling can be acquired as early as the embryonic stem cell stage, and its distribution is dynamic and progressive throughout cell differentiation and tissue development.

## Background

Post-translational modifications of histones play important roles in regulating DNA activities and gene expressions in eukaryotic cells [1-4]. In the nucleus, chromatin forms basic units called nucleosomes which constitute an octamer with eight different histone protein molecules and a 146bp DNA wrapped around it [1]. Each histone molecule has two tails which contain amino acid residues subject to a variety of modifications such as methylation, acetylation, phorsphorylation, and ubiquitination. Such modifications can affect (promote or repress) the accessibility of the DNA to RNA polymerase during gene transcription and thus can activate or silence certain genes [1,3,5]. More importantly, some of such modifications (e.g., the methylation on certain lysine and arginine residues on the N-terminal side) are stable during cell division and considered important epigenetic events. In the past few years, with the rapid progress in high throughput technologies such as microarray and massive parallel sequencing, many exciting new discoveries have been made on studying the genome-wide binding landscapes of different histone modification marks using ChIP-chip and ChIP-seq methods [1,6-11]. In these studies, researchers discovered the combination of different histone modifications mark different chromatin states which often correspond to different genomic annotations such as promoters and enhancers [1,11-13]. Such observations have led to discoveries and predictions of many new regulatory sites on the genome [13,14]. In addition, the ChIP-chip and ChIP-seq technologies enable researchers to investigate the distribution of the histone marks (and other proteins) on the genome at a high resolution, which led to the discovery that the binding patterns of the proteins and histone marks over the genome are also highly informative in predicting genomic functions and annotations [13,15-17]. An interesting discovery along this line is the relationship between H3K4me2 binding patterns and tissue specific genes [17].

H3K4me2 (dimethylation of the lysine residue at 4^th ^position on the N-terminal tail of histone 3) is an important histone mark and has been shown to bind to both gene promoter regions and enhancer regions and its binding is often associated with gene activation. A recent study demonstrated that in human CD4+ T cell and neural tissue, H3K4me2 shows specific binding patterns on the tissue-specific genes in their transcribed regions [17]. In this study, the focus is on a 10kb range of DNA covering 2kb upstream of the transcription starting site (TSS) and 8kb downstream of the TSS for the genes. The H3K4me2 binding patterns over this range for all the genes are then extracted and K-means algorithm is applied to cluster these patterns into five groups. Interestingly, a group distinct itself from others as this group not only shows a bimodal distribution around the TSS, it also uniquely shows high binding quantities (or long tail) over the 8kb downstream of the TSS. Genes in this group are then shown to be highly enriched with tissue specific genes.

However, the recent characterization of histone mark distribution on tissue specific genes was carried out in matured tissue cells, little is known about the dynamic change of histone modification during the cell differentiation and development, neither is it known that at what developmental stage the H3K4me2 marking is acquired. It is generally accepted that the histone modifications play an important role in the cell differentiation and tissue development [18]. Here we are using the public available mouse H3K4me2 ChIP-seq data from different developmental stages of neural cells, namely embryonic stem cells, neural progenitor cells, and whole brain cells [19], and try to address the question that if the same tissue-specific patterns of H3K4me2 present in the neural cells, and if so, how early it emerges and how it redistributes during the developmental process.

## Results

### Identify tissue specific genes in the whole brain tissue

**Figure 1:**
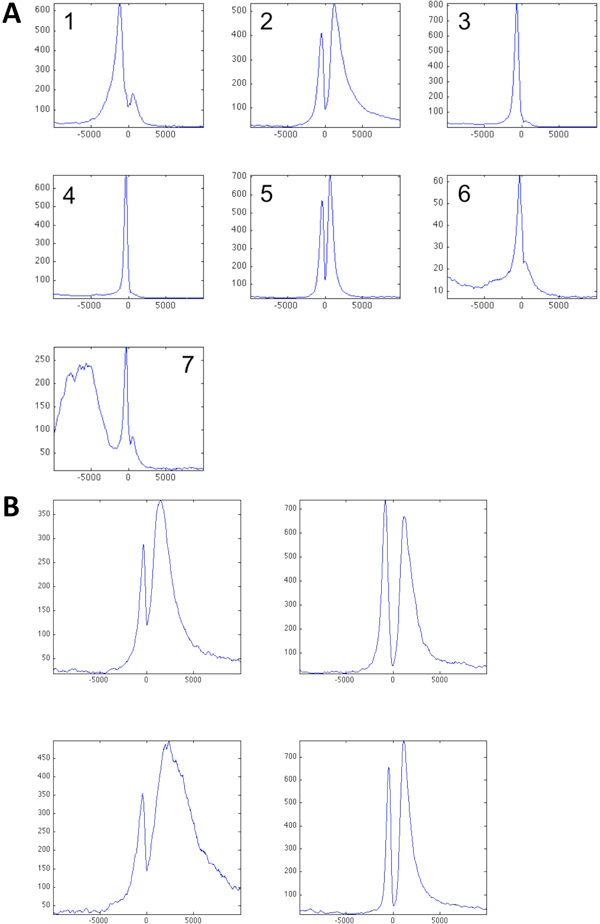
**Average H3K4me2 binding showing clusters from K-means clustering of WB in the extended TSS region.** A: K-means clustering on the entire gene sets, K=7. B: Further K-means clustering within cluster 2. K=4. The cluster on the lower left of Panel B containing 356 genes was further studied. X-axis is the coordinates of the extended TSS region with 0 denoted the TSS. Y-axis is the binding reads counts. The region extends to +/- 10kbp of TSS. WB: whole brain.

K-means clustering of H3K4me2 binding in the ±10kb-extended TSS region of brain dataset identified seven clusters each with distinctive H3K4me2 binding pattern in this region (Figure 1A). Among them, cluster 2 has a prolonged tail of H3K4me2 binding in the transcribed region. The feature of the prolonged tail of H3K4me2 binding in the transcribed region was shown by the previous study [17] as the marks for tissue-specific genes. Therefore, we focused on this cluster of genes for our study of tracing tissue-specific H3K4me2 marks in neural cell differentiation and neutral tissue development. However since the first stage of K-mean clustering is coarse, it was subject to refined clustering (k = 4) to search for the gene subset with more uniformed H3K4me2 binding in this region. The further clustering identified one cluster of 356 genes with substantial H3K4me2 labeling in the transcribed region and a small peak of H3K4me2 upstream of TSS (lower left cluster in Figure 1B). GO enrichment analysis revealed that this cluster of genes are highly enriched neural functions such as transmission of nerve impulse (p-value 1.1E-8) and synapse function (p-value 1.1E-7). In addition, 55% of this gene set are phosphproteins, and participate in various neural system functions, such as synaptic transmission (p-value 1.8E-8), cell-cell signaling (p-value 4.2E-7), construct presynaptic membrane (p-value 8.6E-8), etc. These results are consistent with the finding that heavy presence of H3K4me2 downstream of TSS marks the tissue-specific genes, i.e., brain-specific genes in this case.

### H3K4me2 distributions are dynamic over different stages of development

**Figure 2:**
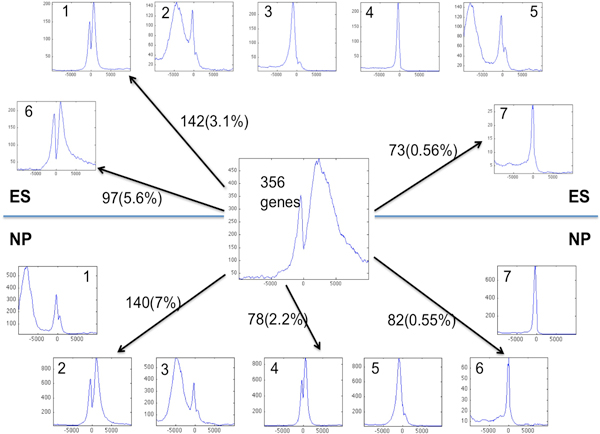
**The redistribution of 356 genes in K-means clustering results of the ES and NP datasets**. The numbers inside parenthesis are the percentage of a specific gene cluster overlapping with 356 gene list. The central plot was the same in the lower left panel from Figure-1B. X-axis is the coordinates of the extended TSS region with 0 denoted the TSS. Y-axis is the binding reads counts.

**Figure 3:**
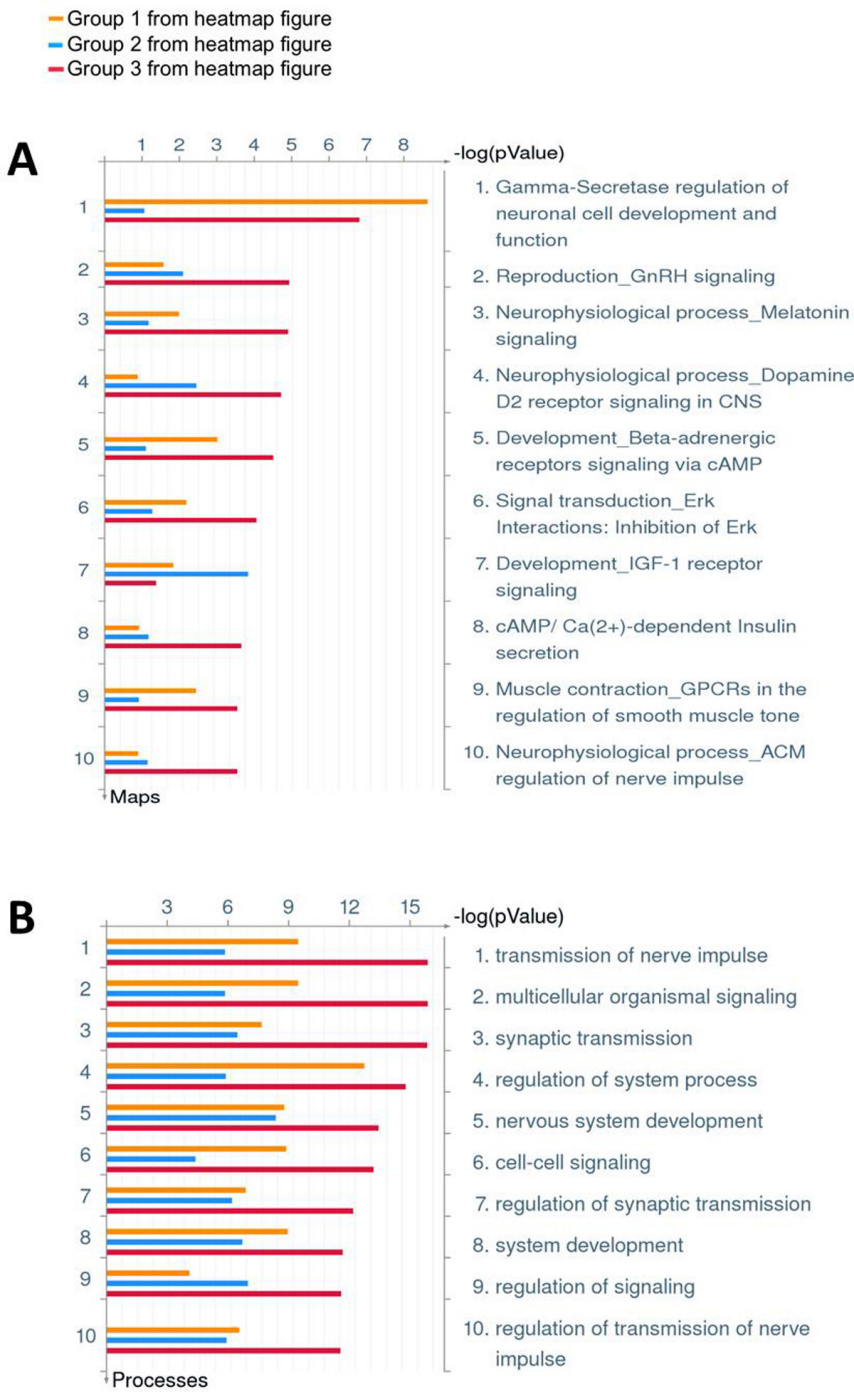
**GO enrichment analysis on group 123 identified from hierarchical clustering of the 356-gene H3K4me2 binding of the combined data**. A: enriched pathways; B: enriched biological processes. The analysis was performed using MetaCore^®^.

**Figure 4:**
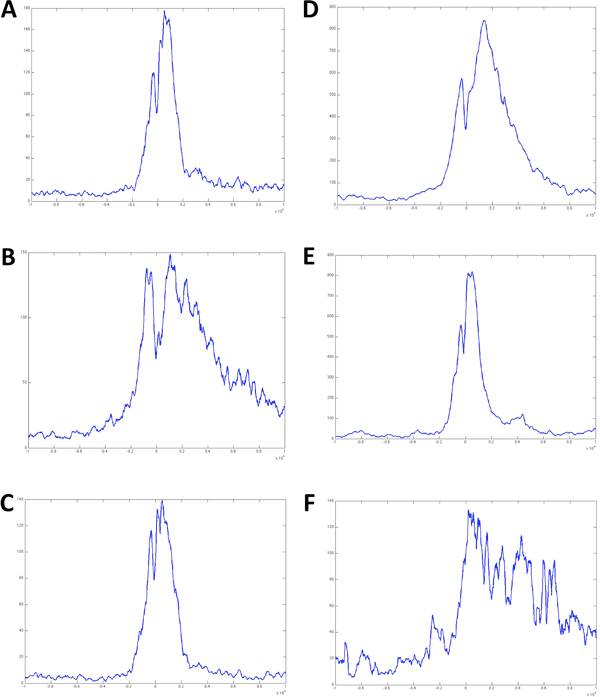
**Average H3K4me2 binding profiles of genes shared by each ES/NP clusters and the 356-gene list**. A: from genes shared by ES cluster 1 and 356-gene list. B: from genes shared by ES cluster 6 and 356-gene list. C: from genes shared by ES cluster 7 and 356-gene list. D: from genes shared by NP cluster 2 and 356 gene list. E: from genes shared by NP cluster 4 and 356-gene list. F: from genes shared by NP cluster 6 and 356-gene list. The region covers +/- 10kbp of TSS. X-axis is the coordinates of the extended TSS region with 0 denoted the TSS. Y-axis is the binding reads counts.

Using the same K-means clustering setting, we further separated ES and NP datasets into different H3K4me2 binding patterns in the extended TSS region (Figure 2). Most of them match the binding profiles of brain tissue, but none of them (except cluster 6 of ES) resembles cluster 2 of brain, which has an elevated H3K4me2 in the downstream of TSS. The above identified 356-gene list overlaps significantly with ES clusters 1 (142), 6 (97) and 7 (73), and with NP clusters 2 (140), 4 (78) and 6 (82) (Figure 2). ES clusters 1, 6 and 7 are enriched with processes and networks involved in nerve system development, such as signal transduction of cAMP (p-value 1.686E-6), neurogenesis (p-value 7.284E-6) and nerve impulse transmission (p-value 1.284E-7) (Figure 3). After checking the average H3K4me2 binding profile in the extended TSS region for those genes shared with 356-gene list (Figure 4), only the cluster 6 of ES (97 common genes) shows H3K4me2 labeling downstream of TSS (panel B in Figure 4), while both the NP cluster 2 (140 common genes) and 6 (82 common genes) show significant H3K4me2 labeling downstream of TSS (panel D and F in Figure 4). Therefore, the tissue-specific mark is spreading to more genes as cells further differentiate into the neural progenitor stage. The NP cluster 6 contains about half of the entire probeset, so further clustering using K-means was carried out, and it reveals a cluster of 194 genes exhibits heavy H3K4me2 labeling downstream of TSS (data not shown). Interestingly, only 14 were found in the 356-gene list. It demonstrated that the H3K4me2 labeling is a progressive yet dynamic process as cells differentiate from embryonic stem cell through neural progenitor till the final matured brain tissue stage. There are groups of genes with constant H3K4me2 labeling, and groups of genes with H3K4me2 levels change throughout development.

### Acquisition of heavy downstream H3K4me2 presence during different stages

**Figure 5:**
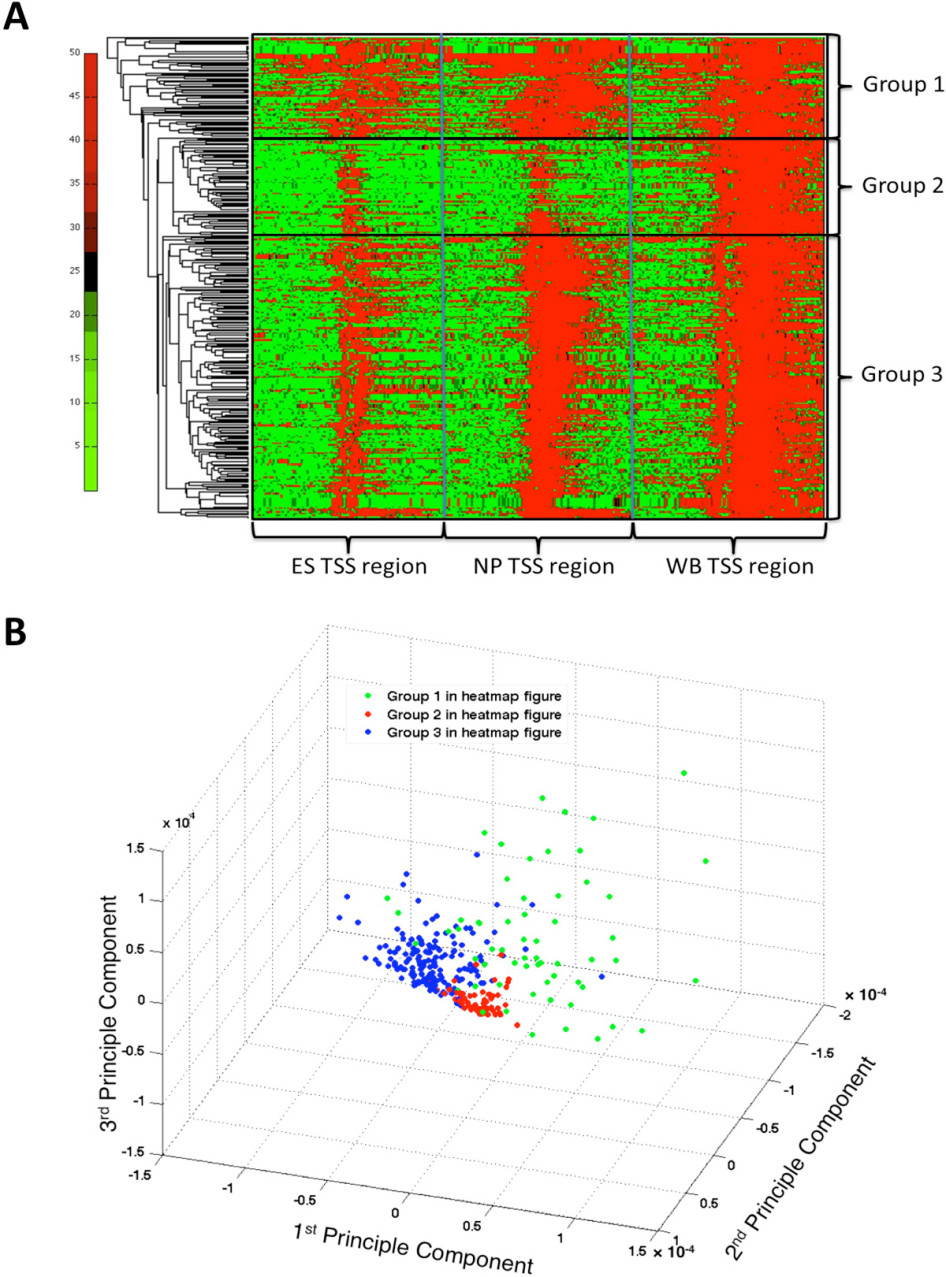
**Hierarchical clustering and Principal component analysis (PCA) result on the combined data from ES, NP and WB for H3K4me2 binding for 356-gene list.**A: Hierarchical clustering. Each row is a range of +/-10kbp on each side of TSS of a gene. The H3K4me2 binding abundance is shown in the color scale. The H3K4me2 binding patterns of the three groups of genes were indicated by the rectangle boxes. The two vertical bars indicate the range of the extended TSS region from each source. B: PCA analysis. The color dots represent different groups from Panel A. The region covers +/- 10kbp of TSS.

**Figure 6:**
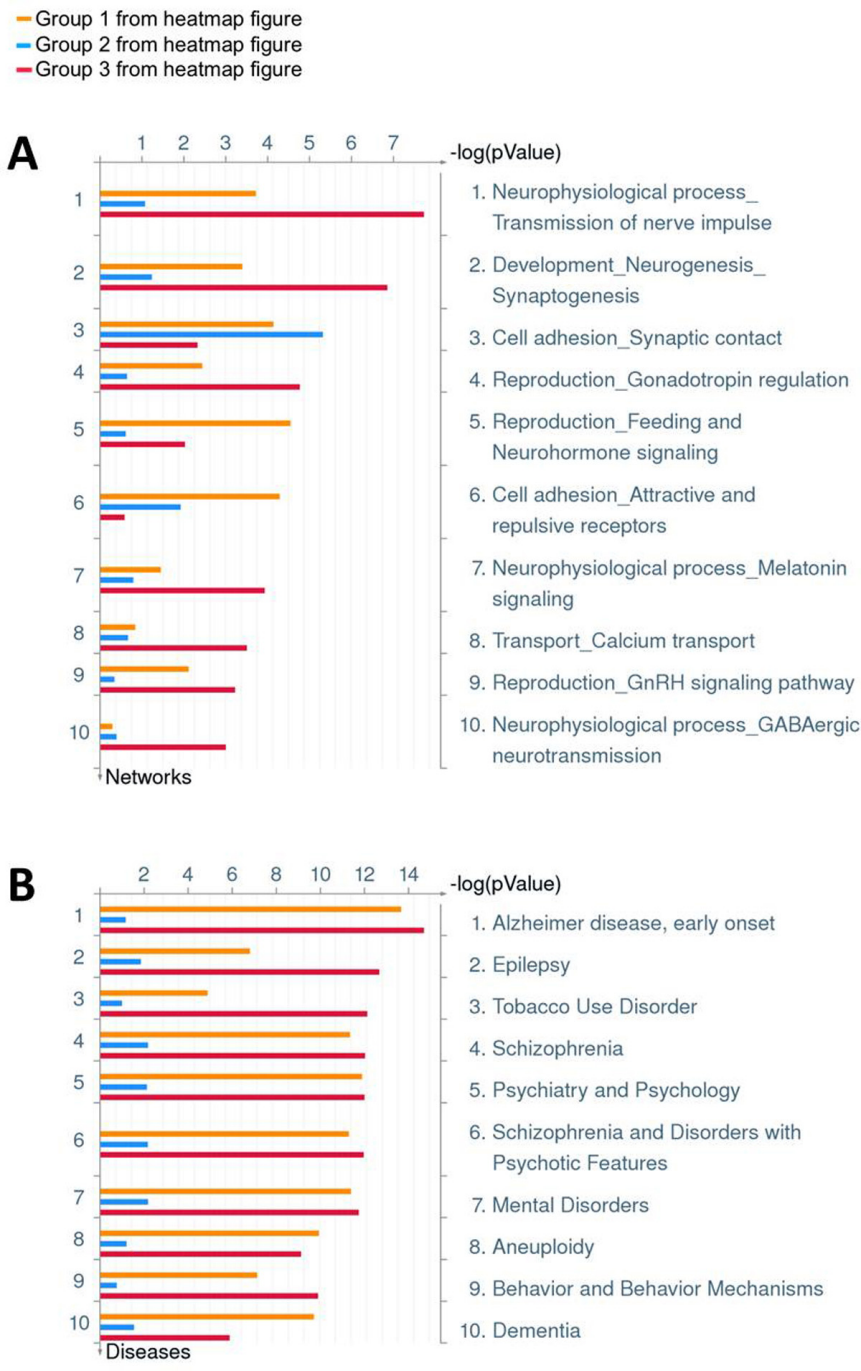
**Network and disease enrichment analysis on group 123 identified from hierarchical clustering of the 356-gene H3K4me2 binding of the combined data**. A: enriched networks. B: enriched diseases. The analysis was performed using MetaCore^®^.

To confirm the above observation, we combined the three H3K4me2 TSS binding profiles from ES, NP and WB data for the 356 genes, and performed a hierarchical clustering based on correlation of the binding pattern in this region. Visually the results revealed clearly three distinct H3K4me2 labeling groups as the downstream presence of H3K4me2 of TSS as the development of neural system proceeds from ES through NP to the mature brain stage (Figure 5A). The principle component analysis (PCA) on the same data also confirmed that there exists three well-separated group of genes (Figure 5B). Group 1 contains 74 genes with constant H3K4me2 labeling from ES through NP to matured brain. This group has more diverse H3K4me2 binding features than the other two groups as shown in Figure 5B. GO enrichment analysis shows that this group is enriched with γ-secretase regulation of neuronal cell development and function (p-value 2.313E-9) (Figure 3A). Group 2 contains 73 genes with minimal H3K4me2 labeling in ES and NP stage, but gets heavy H3K4me2 labeling in the matured brain tissue. This group is significantly enriched with cell adhesion of synaptic contact (p-value 5.272E-6) (Figure 6A), and the H3K4me2 binding feature is quite uniform as shown to form a tight cluster in Figure 5B. Group 3 is the largest one containing 209 genes with minimal H3K4me2 labeling in ES stage, but gradually gains H3K4me2 labeling as the cells differentiate into NP cells then mature as brain tissue. It is enriched with several neural transmitter signaling pathways, such as GnRH, melatonin, dopamine D2 receptor, β-adrenergic receptors signaling pathway, and participates in processes such as nerve impulse transmission (p-value 1.337E-16) and multicellular organismal signaling (p-value1.337E-16) (Figure 3B) as well as synaptogenesis (p-value 1.451E-7) (Figure 6A). Both groups 1 and 3 are highly enriched with genes involved in several neural degenerative and neurological diseases, such as Alzheimer early onset, epilepsy, tobacco use disorder, schizophrenia, and brain dementia, while cluster 2 genes are the least enriched with such disease genes (Figure 6B).

## Discussion

In this paper, we applied detailed clustering analysis on the H3K4me2 distribution patterns around the TSS regions of genes in the whole genome. Our analysis confirmed the previous report that heavy presence of H3K4me2 in the downstream of TSS marks tissue specific genes. However, more importantly, we have shown that such patterns are not constant. Instead, redistribution of H3K4me2 occurs dynamically in all stages of tissue development.

In addition, our results shed light on understanding the acquisition of tissue specificity during the developmental process. For the 356 brain tissue specific genes, their H3K4me2 distribution patterns around the TSS regions over the three developmental stages can clearly be divided into three major groups. The GO enrichment analysis of the three groups of genes indicates that a small portion of neural system-related development and functions was activated or prone to be activated as early as the embryonic stem cell stage, as shown by the H3K4me2 marks on a small group of genes, whereas most of the neural system-related functions were developed or scheduled to develop through the neural progenitor cell stage, as indicated by the H3K4me2 marks on a larger set of genes in the NP stage. There is a group of genes acquired heavy H3K4me2 labeling downstream of TSS only when the matured brain cell is formed, and consistently cell adhesion activity such as synapse contact formation become the dominate process. This H3K4me2 re-distribution pattern fits very well with the developmental process of neural system.

An interesting observation about these three groups is the gene involvement in neural degenerative and neurological diseases as well as mental disorders including Alzheimer early onset, brain dementia, and schizophrenia. Most of genes associated with these diseases are in the group 1 (with heavy H3K4me2 in ES stage) or group 3 (with heavy H3K4me2 acquired in NP stage). In addition, GO function analysis also suggests that groups 1 and 3 genes are more involved in essential neural functions such as transmission of nerve impulse and synaptic transmission while the group 2 (with heavy H3K4me2 only acquired in the latest stage) are more involved in developing and maintain synapse structures as a mature brain function. These observations imply that these disease-related genes are more involved in the essential neural tissue functions than in the advanced brain activities.

Besides H3K4me2, many other histone marks as well as epigenetic events such as DNA-methylation are also involved and more insight about the differentiation process can be gained with more data available. In addition, the brain tissue data in this study is obtained from whole brain sample even though it is well known that different anatomical and functional regions of the brain have distinctive gene expression patterns and more refined analysis of region and function specific genes could be identified using similar approach as in this paper.

## Conclusions

In summary, we investigated the process of the H3K4me2 mark redistribution during tissue specificity development for mouse brain tissue. Our analysis confirmed the previous report that heavy labeling of H3K4me2 in the downstream of TSS marks tissue specific genes. These genes are not only highly enriched with neural system functions and pathways, but also highly involved in CNS diseases. Furthermore, we have shown that such labeling can be acquired as early as the embryonic stem cell stage, and its distribution is dynamic throughout cell differentiation and tissue development. The distribution of H3K4me2 on neural function related genes follows three distinctive patterns: a group of genes contain constant heavy H3K4me2 marks in the gene body from ES through NP to matured brain tissue stage; another group of gene have little H3K4me2 marks until cells mature into brain cells; the majority of the genes acquired H3K4me2 marks in the NP stage, and gain heavy labeling in the matured brain cell stage. GO enrichment analysis also revealed corresponding GO terms that fit in the scenario of each cell developmental stages.

## Methods

Three H3K4me2 ChIP-seq datasets are downloaded from NCBI Gene Expression Omnibus with accession number GSE11172 including murine embryonic stem (ES) cells, ES-derived neural progenitor (NP) cells, and whole brain (WB) tissues [19]. The 36-bp short reads were generated using Illumina GA sequencing platform. In our analysis, we extended the short reads by 200bp, and histograms of the extended reads counts over a 20-kb TSS region (+/- 10kb of TSS) for each entry from the RefSeq mm8 database (obtained from UCSC website) were computed using bin size 20bp. Unsupervised clustering method K-means was used to cluster each individual TSS region profiles from the three origins (ES, NP and WB) using k = 7, repeats = 50, distance = square Euclidean as implemented in Matlab. We empirically selected k = 7 based on visual inspection of clustering results from different values of k.

In WB tissue, the cluster with an elevated H3K4me2 marking downstream of TSS was further divided into four clusters using K-means (k = 4, repeats = 50, distance = square Euclidean) to obtain a cluster of 356 genes with highly elevated H3K4me2 labeling downstream of TSS. This set of genes was compared with clusters obtained from ES and NP samples, and largely overlapped genes and clusters were further subject to GO enrichment analysis using the MetaCore software as well as the NIH DAVID tool.

At the same time, the combined TSS profiles of the 356 genes from all three origins were clustered using hierarchical clustering in Matlab and three clusters were selected based on visual inspection: the cluster with constant H3K4me2 labeling from ES through NP to matured brain; the cluster with minimal H3K4me2 labeling in ES and NP stage, but gets heavy H3k4me2 labeling in brain tissue; and the cluster with minimal H3K4me2 labeling in ES stage, but gradually gains H3K4me2 labeling as the cells differentiate into NP cells and mature as brain tissue. Each cluster of genes was subject to gene set enrichment and network analysis using bioinformatics software MetaCore™ by GeneGo (http://www.genego.com/metacore.php), as well as Database for Annotation, Visualization and Integrated Discovery (DAVID, http://david.abcc.ncifcrf.gov/). Principal Component Analysis (PCA) was performed on the same combined data using Matlab code.

## List of abbreviations

H3K4me2: dimethylation of lysine 4 residue on histone 3; ES: embryonic stem; NP: neural progenitor; WB: whole brain; GO: gene ontology; TSS: transcription start site; GnRH: Gonadotropin-releasing hormone; ChIP-seq: chromatin immunoprecipitation sequencing; CNS: central nerve system; PCA: principal component analysis

## Competing interests

The authors declare that they have no competitng interests.

## Authors' contributions

JZ performed all the analysis and wrote the manuscript. JDP participated in the discussion and manuscript editing. KH designed the study and participated in the writing of the manuscript.
